# Therapeutic Application of rTMS in Atypical Parkinsonian Disorders

**DOI:** 10.1155/2021/3419907

**Published:** 2021-12-23

**Authors:** Chrysi Petsani, Athina-Maria Aloizou, Vasileios Siokas, Lambros Messinis, Eleni Peristeri, Christos Bakirtzis, Grigorios Nasios, Efthimios Dardiotis

**Affiliations:** ^1^Department of Neurology, University Hospital of Larissa, School of Medicine, University of Thessaly, Larissa, Greece; ^2^School of Psychology, Lab of Cognitive Neuroscience, Aristotle University of Thessaloniki, Greece; ^3^Department of English Studies, Aristotle University of Thessaloniki, Greece; ^4^Multiple Sclerosis Center, 2nd Department of Neurology, AHEPA University Hospital, Aristotle University of Thessaloniki, Thessaloniki, Greece; ^5^Department of Speech and Language Therapy, School of Health Sciences, University of Ioannina, Ioannina, Greece

## Abstract

The terms atypical parkinsonian disorders (APDs) and Parkinson plus syndromes are mainly used to describe the four major entities of sporadic neuronal multisystem degeneration: progressive supranuclear palsy (PSP), corticobasal degeneration (CBD), multiple system atrophy (MSA), and dementia with Lewy bodies (LBD). APDs are characterized by a variety of symptoms and a lack of disease modifying therapies; their treatment thus remains mainly symptomatic. Brain stimulation via repetitive transcranial magnetic stimulation (rTMS) is a safe and noninvasive intervention using a magnetic coil, and it is considered an alternative therapy in various neuropsychiatric pathologies. In this paper, we review the available studies that investigate the efficacy of rTMS in the treatment of these APDs and Parkinson plus syndromes. *Τ*he majority of the studies have shown beneficial effects on motor and nonmotor symptoms, but research is still at a preliminary phase, with large, double-blind studies lacking in the literature.

## 1. Introduction

### 1.1. Atypical Parkinsonian Disorders/Parkinson plus Syndromes

Progressive supranuclear palsy (PSP), multiple system atrophy (MSA), corticobasal degeneration (CBD), and Lewy body dementia (LBD) are the most important entities of the neurodegenerative disorders consisting the atypical parkinsonian disorders (APDs) or the so-called “Parkinson Plus” syndromes. The clinical phenotypes of these syndromes present great heterogeneity, as a result of the different underlying pathophysiological mechanisms. These disorders manifest as an atypical parkinsonian syndrome with symmetric distribution, rapid deterioration, and poor response to medications (levodopa or other dopaminergic agonists). In addition to parkinsonism, other atypical clinical symptoms are also present, such as supranuclear gaze palsy, asymmetrical apraxia, early postural instability, early dementia, and symptoms from the autonomic system [1, 2]. APD sare subdivided into “synucleinopathies” and “tauopathies,” based on the abnormally accumulated protein that contributes to the neurodegenerative damage (i.e., a-synuclein or Tau). MSA and LBD are considered synucleinopathies, while PSP and CBD are tauopathies. Although APDs are rarer disorders than PD, the differential diagnosis is very important, since disease deterioration and functional deficits usually appear earlier than in PD [3], and classic PD therapies are only partially beneficial.

The most common of the atypical parkinsonian syndromes is PSP and is usually difficult to distinguish from PD. Early postural instability and falls, accompanied by an akinetic rigid syndrome and ocular dysfunction, are the most common and typical expressions of this disorder [2, 4, 5]. These symptoms characterized the phenotype of PSP now called Richardson's syndrome (PSP-RS). Until the 2017 update, the criteria for the clinical differential diagnosis of PSP had remained unchanged since 1990 [4]. The 2017 update emphasized that PSP encompasses a number of different clinical phenotypes and outlined ten, with Richardson's syndrome (PSP-RS) being only one among those ten [6]. Pathophysiologically, PSP features an overexpression of a particular tau protein isoform, the 4R-tau, which contains four microtubule-binding repeat domains [7–9]. The tufted astrocyte is the most common pathological abnormality in PSP, while neurofibrillary tangles (NFTs) and coiled bodies usually contribute to the pathology as well [10]. The different localization of tau protein accumulation drives the different clinical phenotypes. Brainstem pathology is expressed with pure akinesia, while cortical pathology creates a focal cortical syndrome [7]. Studies have revealed an important involvement of cerebellar structures in PSP pathology, and especially the dentate nucleus, despite cerebellar signs in this disorder being rare [4].

MSA is a neurodegenerative disorder, manifesting with parkinsonism, cerebellar ataxia, and autonomic dysfunction [11]. Based on the predominant symptoms, two main MSA phenotypes are distinguished: the MSA-C with predominant cerebellar symptoms and the MSA-P with predominant parkinsonian manifestations [11, 12]. Sleep changes (particularly R*ΕΜ* sleep behavior disorders), autonomic failure, and respiratory dysfunction are common in both subtypes and can precede motor symptoms even for years [13]. MSA, as already mentioned, belongs to the synucleinopathies, and its pathology is characterized by glial cytoplasmatic inclusions formed by fibrillated *α*-synuclein proteins in the striato-nigral and olivo-ponto-cerebellar areas [14].

CBD is a rare degenerative neurological disorder pathologically characterized by asymmetrical cortical brain atrophy, usually more pronounced at the frontoparietal regions, combined with degenerated basal ganglia. The term CBD describes the pathology of a disease which usually but not always coexists with clinical symptoms encompassed by the corticobasal syndrome (CBS). The CBS phenotype usually includes asymmetric hand dysfunction, bradykinesia, dysphagia, tremor, rigidity, dystonia, and gait and postural instability in the spectrum of motor symptoms, while cognitive impairment, visuospatial deficits, and apraxia constitute the nonmotor spectrum [15].

Finally, LBD comes after Alzheimer's disease (AD), as the second most frequent neurodegenerative dementia, encompassing dementia with Lewy bodies (DLB) and Parkinson's disease dementia (PDD) [16]. The pathological characteristic of this disorder is the aggregation of a-synuclein, creating the so-called Lewy bodies. Parkinsonism, cognitive impairment, serious behavioral disorders, vivid and recurrent hallucinations, and sensitivity to antipsychotic medications are the most common and typical symptoms [17].

Another common element of these entities is the absence of disease-modifying drugs (DMDs) or other treatment options that are effective in this regard [16]. The treatment of APDs remains largely symptomatic, for example, with botulinum injections when dystonia manifests [18], while levodopa is either ineffective or effective for a short period of time [19], so no amelioration in parkinsonism symptoms can be easily achieved. It is thus evident that safe and effective treatment options are urgently needed. A new research field that has been gaining more ground in this direction is the application of transcranial magnetic stimulation (TMS).

### 1.2. TMS Principles

In 1985, TMS was firstly introduced in the group of noninvasive brain stimulation techniques [20]. TMS uses a magnetic coil targeting the scalp and producing a high-intensity pulse, which stimulates neurons. Depending on the exact protocol and the different coil parameters, the stimulation of the neurons can vary, giving way to many different intervention potentials [21].

In pathophysiological studies, single and paired stimuli are usually applied, contrary to studies investigating the therapeutic use of TMS, which apply a series of repetitive stimuli [repetitive TMS (rTMS)]. rTMS applied at set frequencies or patterns can alter cortical excitability, lasting long after the end of the stimulation [22]. rTMS can induce long-lasting changes through its effect on blood circulation within the CNS, neuronal metabolism, and excitability of the cortex directly receiving the stimulation and of areas connected to the target of the stimuli [22–24]. In general, the stimulation of the brain modulates the plasticity of the cortex. These changes are induced via long-term potentiation (LTP) and long-term depression (LTD) [22]. Frequency, duration, and intensity are some of the basic parameters which characterize a stimulation protocol, and its effects can be either excitatory or inhibitory. *Η*igh frequency rTMS (HF-rTMS) (>1 Hz) induces LTP and increases cortical excitability, while the application of low-frequency stimulation (LF-rTMS) (≤1 Hz) produces LTD and a decline in cortical excitability [25].

Additionally, rTMS protocols can be further subdivided into simple protocols with identical interstimulus intervals (ISI) between the pulses and patterned protocols with different ISIs. Theta burst stimulation (TBS) belongs to the patterned group. TBS modulates cerebral cortical function, via HF-rTMS that mimics the theta brain waves, consisting of three 50 Hz pulses every 200 ms. TBS application includes two different protocols, the intermittent TBS (iTBS) and the continuous TBS (cTBS); the former increases cortical excitability while the latter decreases it [26].

The use of TMS has been associated with some adverse effects. Transient headaches and scalp discomfort are the most common, and are linked to the activation of scalp pericranial muscles [27]. Furthermore, changes in mood (cases of inducted mania), burns of the scalp, and seizures are the most severe side effects [27, 28]. However, these adverse events are extremely rare, so rTMS is generally considered a safe treatment modality.

rTMS has been considered to be a therapeutic option for many pathologies, such as depression, migraine, and epilepsy [29–31], and even neurodegenerative conditions with cognitive sequelae, such as Alzheimer's disease [32]. In addition, rTMS has been extensively studied in PD, showing positive effects in motor and nonmotor symptoms and in therapy complications [33]. This review aims to summarize the available literature concerning the therapeutic intervention of rTMS in APDs and to discuss its future applications. Based on our knowledge, it is the first review to investigate the application of rTMS in the entirety of the APDs. Tables 1 and 2 summarize all the available clinical trials studying the therapeutic application of rTMS in PSP and MSA patients, respectively, while studies involving rTMS in CBD and DBL can be found in Table 3.

## 2. Supranuclear Palsy (PSP)

### 2.1. Cerebellar Stimulation

An increasing amount of evidence has supported the involvement of the cerebellum in PSP pathophysiology. Tau isoforms have been shown to accumulate in the cerebellum and lead to reduce cerebellar volumes [7]. In addition, TMS studies have detected an impairment of functional connectivity to the pathway of the contralateral primary motor cortex (M1) and the cerebellar hemispheres [cerebellar brain inhibition (CBI)] [42]. Levodopa can only partially and temporarily alleviate some of the PSP symptoms, such as akinesia and rigidity [43], with postural instability remaining an important problem. Based on these considerations, a line of studies has explored the effectiveness of cerebellar rTMS in PSP.

The first published open-label trial using TBS over the cerebellum of PSP patients was conducted by Brusa et al. [34]. Ten PSP-RS patients entered the study and were then clinically evaluated based on the PSP Rating Scale (PSP-RSc). Two control groups, one of PD patients and another of healthy age-matched subjects, were also enrolled. The cerebellar iTBS protocol (3 50 Hz pulses, repeated at a rate of 5 Hz, 20 trains of 10 bursts in 8 s intervals, 600 pulses, 80% of AMT intensity) was applied bilaterally to the cerebellum of all subjects for 10 days. Before and after the iTBS application, functional connectivity between the cerebellum and the contralateral M1 (CBI), intracortical facilitation (ICF), short intracortical inhibition (SICI), and short latency afferent inhibition (SLAI) in the contralateral M1 were measured. Resting state functional magnetic resonance (rs-fMRI) was performed, and the PSP-RSc was administered. After the iTBS treatment, all PSP patients significantly improved in dysarthria, and 2 out of 10 patients reported a significant amelioration in gait. Only CBI metrics improved upon stimulation. This study concluded that PSP patients after cerebellar iTBS showed some clinical improvement and a parallel enhancement in functional connectivity between the hemisphere of the cerebellum, the caudate nucleus, and the brain cortex. However, a placebo effect could not be excluded due to the open-label trial design.

The efficacy of rTMS over the cerebellum in PSP was also investigated in a sham-controlled case study by Dale et al. [35]. They performed CBI assessments with neuronavigation before and after cerebellar HF-rTMS or sham TMS in two patients with PSP, collecting posturography data and speech samples before and after the intervention. Quality of speech was assessed via reading a standard passage, and pace of speech, articulatory difficulty, and article and phonemic errors were noted. The exact rTMS protocol included 4,000 pulses delivered over the cerebellum (10 Hz, 90-110% of Resting Motor Threshold (RMT) intensity), with 10 days of active treatment and 10 days of sham, separated by a month. After treatment, CBI increased by 50% in subject 1 and by 32% in subject 2, while stability and speech also presented an improvement. However, a different form of sham stimulation was applied in the two subjects. Patient 2 received sham stimulation from a coil with a magnetic-blocking spacer, whereas patient 1 had the same spacer with extra superficial electrical stimulation. This superficial stimulation could not produce the same burning sensation in the posterior head and neck area as the active one, so patient 1 was able to guess that this was indeed a sham condition. This unexpected placebo effect in patient 1 means that these results must be taken into consideration with even greater caution.

Pilotto et al. [36] conducted a trial which overcame the placebo effect problem. They designed a double-blind study controlled with sham stimulation and assessed postural stability via mobile health technology. Twenty probable PSP patients were included. All subjects received both real and sham TMS intervention in two different sessions, with an interval of two weeks. The exact protocol included repetitive cerebellar TBS (3 50 Hz pulses repeated at a rate of 5 Hz, 20 trains of 10 bursts in 8 s intervals, 600 pulses, 80% of RMT intensity). The sham stimulation was applied with a coil attached by a spacer so that all the circumstances were identical to the real one, and the subjects could not differentiate the two conditions. Clinical evaluation was conducted on all patients before and after each stimulation, with the Tinetti test, the Timed Up and Go test, the Short Physical Performance Battery (SPPB), and the Functional Reach test (FR). Furthermore, four different tasks, with a duration of 30s each, contributed to the assessment of static balance, also conducted before and after each stimulation. These tasks included tandem and semitandem stance with eyes open and closed, and additionally, an inertial sensor unit (IMU) located over the third lumbar segment of spine, processing and calculating acceleration signals, was also used. Active stimulation was associated with greater stability, during all tasks, contrary to the sham condition. Significant improvement in area, velocity and acceleration, and jerkiness of sway, as denoted from IMU extracted parameters, was detected after active stimulation only.

What can be easily deduced from these studies is that cerebellar rTMS holds promise in tackling postural stability and speech impairment in PSP patients. However, the patient numbers remain small, and as such, bigger and better designed clinical trials are needed to confirm its efficacy and determine the most appropriate protocol.

### 2.2. Motor Area Stimulation

Motor cortex disinhibition has been shown to be a predominant feature in PSP pathology [44]. RTMS has already been considered as a possible therapy method for parkinsonism in PD, and its therapeutic contribution to other similar disorders such as PSP is under investigation, especially regarding axial rigidity and falls, cardinal symptoms of PSP.

The first pilot study exploring the efficacy of rTMS application over the motor cortex in PSP patients was carried out by Santens et al. [37]. In this study, 6 PSP-RS patients were enrolled. The subjects received HF-rTMS (10 Hz, 80% of MT intensity) of 1000 pulses targeting the lower limb motor area for 5 consecutive days. Clinical evaluation was conducted at baseline and after the last stimulation on all patients, according to the Clinical PSP-RSc. The total score of PSP-RSc improved in five of the patients after the stimulation, with the most prominent effect shown on the gait/midline symptoms. A subjective improvement of overall function and mobility was reported from the subjects, albeit lasting for only 2-3 days. These findings suggest a potential benefit of rTMS in PSP patients, especially for gait and midline symptoms. Nevertheless, the validity of these results is questioned due to the small cohort and the absence of sham stimulation.

Nishida et al. [38] investigated the efficacy of rTMS in 6 PSP-RS cases and one PSP-pure akinesia with gait freezing (PSP-PAGF) patient. Evaluation at baseline and after the stimulation was carried out on all subjects via the PSP-RSc. Real HF-rTMS (5 Hz, 110% of RMT intensity) of 500 pulses over the supplementary motor area (SMA) was applied for 10 days. The 10 trains of each session were equally shared between the two hemispheres. The results showed that rTMS increased PSP-RSc scores by 7 points. However, only total PSP-RSc scores significantly improved, contrary to each subitem of the scale, which did not show a significant individual change. Sham controlled stimulation was not included in the trial, and as such, a placebo effect could not be excluded.

Major et al. [39] studied the effects of rTMS on the motor symptoms of a PSP patient using goniometry and dynamometry [39]. The case subject was a 65-year-old man with a dominant right hand. LF-rTMS (1 Hz, 80% of RMT intensity) was applied, with a 20 min duration per day, for five days consecutively, over the motor cortex bilaterally. Mechanography evaluation included a goniometer, recording the angles in 15 simple movements, and a dynamometer measuring muscle strength. A significant increase in range motion and muscle strength was reported after the stimulation.

Collectively, these studies show that rTMS over the motor areas can provide beneficial effects on motor symptoms in PSP patients. However, the small cohorts, the absence of sham stimulation control, and the possible placebo effect question the generalization of the reported results. Furthermore, trials including all PSP phenotypes (not only PSP-RS) should be conducted. The results are promising, but still, more trials are needed to evaluate their persistence and reproducibility. Additionally, evidence is stronger for HF-rTMS, but small-scale evidence of LF also being effective, such as the aforementioned case report, raise questions regarding the underlying mechanisms in PSP, and what researchers will need to target in the future, and how.

### 2.3. Dorsolateral Prefrontal Cortex Stimulation

Prefrontal cortex abnormalities are thought to be the pathophysiological source of depression in PSP patients [45]. Following this line of thought and based on the fact that rTMS over this area has received strong recommendation for treating major depression in the latest guidelines [50], Boulogne et al. [40] applied LF-rTMS (1 Hz, 120% of RMT intensity) targeting the right dorsolateral prefrontal cortex (DLPFC) of a 62-year-old PSP male patient with treatment-resistant major depression. The subject was neurologically and psychologically examined and evaluated using the PSP-RSc and the Montreal cognitive assessment (MoCA), along idea number of other psychiatric scales, namely, the State-Trait Anxiety Inventory (STAI), the Montgomery Asberg Depression Rating scale (MADRS), the Lille Apathy Rating Scale (LARS), and the Global Assessment of Functioning (GAF) Scale, all administered at baseline and after the rTMS intervention. Except for hydroxyzine administration upon serious anxiety symptoms, no other treatments were applied. The researchers observed an improvement in depressive symptoms and apathy after rTMS application; in greater detail, the MADS and STAI scores decreased, while the LARS and GAF scale scores increased after rTMS. This case study shows that rTMS over right DLPFC may relieve depression and contribute to a better life quality of PSP patients, though this remains a sole case report.

Regarding language impairments, Madden et al. [41] published a case report, indicating that stimulation targeting the left DLPFC in PSP patients can produce benefits regarding language functions. The technique of noninvasive stimulation of brain applied on the PSP patient was not TMS but transcranial direct current stimulation (tDCS). The subject studied was a male PSP patient with speech deficits such as declined verb fluency and speech production. A group of language exercises was used to evaluate the patient's language production at baseline and after sham or active application of tDCS targeting the left DLPFC. After each intervention, a different group of exercises was used to avoid any practice effect. This protocol was repeated four times, and the patient was blind to the stimulation status, real or sham. Comparison of speech production effects, between the groups of real and sham intervention, showed that the patient benefited from tDCS in phonemic fluency and action naming.

Taken together, these two cases insinuate that LF-rTMS targeting the right DLPFC can be safe and beneficial for PSP patients with major depression resisting treatment, and that noninvasive brain stimulation over left DLPFC in PSP patients can improve language deficits, although the case report applied tDCS.

## 3. Multiple System Atrophy (MSA)

### 3.1. Cerebellar Stimulation

MSA patients usually present with defective movement control, which stems from cerebellar dysfunction and damage in cerebellar neural pathways [51]. In the cerebellum-M1 circuit, the Purkinje cells inhibit the cerebellar dentate nuclei, which normally induce excitatory effects on M1 area via the ventral thalamus [52]. Degeneration and atrophy of the cerebellum in MSA means Purkinje cell loss, indicating a disinhibition of the dentate nucleus and its excitatory effect [53], becoming a target in rTMS studies.

Liu et al. [46] studied the therapeutic outcome of rTMS on controlling motor movements and spontaneous brain activity in MSA patients [46]. This study enrolled 9 subjects with MSA, who received daily sessions of HF-rTMS (5 Hz, 100% of RMT intensity) for 5 days. The stimulation coil targeted the M1 cortex bilaterally and the right and left lateral cerebellum sequentially. The Unified Multiple System Atrophy Rating Scale (UMSARS) was used for motor control assessment at baseline and within 3 days after the stimulation. Resting-state brain network activity was assessed via fMRI. After the rTMS sessions, improved motor control was found in 7 patients, compared to baseline. In addition, the resting-state complexity of the motor cortex showed an increase after stimulation in 6 patients. The researchers also noticed that the change in motor scores correlated with the change noted in motor network resting-state complexity. This study presented as rationale that multifocal interventions have provided beneficial results in the setting of PD and applied a combined intervention as well. However, whether the noted results stem from stimulation of the cerebellum or the motor cortex or both cannot be deduced from this study. Additionally, the interaction of the simultaneous stimulation needs to be assessed; one cannot exclude a possibility of the two stimulations counteracting each other and attenuating the improvement.

A TMS study by Celebi et al. [54] reported impairments in cognitive functions that correlated with short-latency afferent inhibition (SAI) in MSA patients. SAI is a neurophysiological tool that assesses motor cortex excitability modulation and also corresponds to the inhibition of brain cortex via the cholinergic system [22, 55]. With this background, Yildiz et al. [47] investigated the alteration of cerebellar-cortical interactions in MSA-C patients after cerebellar rTMS intervention. Twelve MSA-C patients, 5 AD patients, and 9 healthy controls entered the study. All subjects were cognitively evaluated with a series of neurophysiological tests. Attention and spatial working memory were evaluated with a simple computerized reaction time (RT) task. Six hundred pulses of LF-rTMS (1 Hz, 90% of RMT intensity) were applied, targeting the lateral cerebellum (ipsilateral to the side recording the motor evoked potential). The study included two different sessions in the same day. Firstly, RT and SAI were evaluated with simple TMS, while during the second session, rTMS was applied, and RT and SAI were reevaluated within 10 minutes from the stimulation. The study found that cerebellar rTMS provoked an important improvement in SAI deficits only in the MSA-C patients. Additionally, regarding the RT, there was a significant improvement in post-rTMS RT values of the MSA-C patients in contrast with the pre-rTMS RT values but not in the healthy control subjects. This study indicates that rTMS over the cerebellum influences SAI, inducing changes in cognitive functions, and may thus be a promising therapeutic approach for MSA patients.

In summary, the few available studies show that rTMS over the cerebellum acts on the abnormal cerebellar-cortical inhibitory neuronal connections of MSA patients. Different protocols with both high and low frequency cerebellar rTMS both seem to induce clinical improvement in MSA patients, which needs to be cleared in future studies, especially double blind studies with larger cohorts and patients with pure cerebellar syndromes. Additionally, assessing the duration of the positive effects also needs to be addressed, by including assessment sessions surpassing the initial week after the intervention.

### 3.2. Motor Area Stimulation

Chou et al. [48] conducted a double-blind, controlled with sham rTMS study assessing HF-rTMS over the left M1 in MSA. Twenty-one right-handed MSA patients were randomly categorized into a real or sham rTMS group. At baseline, all subjects were evaluated for their motor functions using the UMSARSII and received a resting-state fMRI. The rTMS intervention protocol included 10 HF-rTMS sessions (5 Hz, 110% of RTM intensity) of 1000 pulses targeting the left M1, over a span of 2 weeks, one session per day for five days in each week. After the 5th day of intervention, a midstimulation evaluation with the UMSARSII was conducted. At the end of all sessions, all patients received a resting-state fMRI and another UMSARSII assessment. The sham group followed the same protocol but with the coil positioned over the scalp with the back inactive surface. Motor symptoms were significantly improved (decreased UMSARSII) only in the real rTMS group. The resting-state fMRI data investigated differences between the real and sham rTMS application, before and after the rTMS intervention. A set of 47 functional connections was found to be significantly changed in the real rTMS group after the intervention. In addition, when examining the correlation of these brain link alterations and the motor symptoms improvement, a significant association for 10 of these connections was found. None of these correlations were reported for subjects that received sham intervention. This study suggests that HF-rTMS targeting the left M1 produces an improvement of motor symptoms by modulating specific brain functional connections.

The same team also conducted another study investigating the therapeutic outcome of rTMS targeting the left M1 of MSA patients [49]. They enrolled 15 right-handed MSA patients, 7 of which received the treatment and 8 consisted the controls. Additionally, a group of 18 healthy controls subjects, matched on age and sex, was prospectively included. At baseline, all MSA patients were assessed for their motor deficits, with the UMSARSII. The experimental procedure consisted of two fMRI sessions, before and after 10 sessions of HF-rTMS (5 Hz, 110% of RMT intensity) targeting the left M1, over 2 weeks, one session per day for 5 days per week. During fMRI scanning, a tapping exercise was performed. RTMS was not applied to the healthy controls, and fMRI examination was conducted only once. Patients in the sham group followed the same protocol but with the coil touching the scalp from the inactive back side. After the 5th rTMS session, a midstimulation evaluation with the UMSARSII was conducted. At the end of all stimulations, all patients received a resting-state fMRI and a final motor assessment with the UMSARSII. After rTMS treatment, only patients receiving active stimulation showed significant improvements in their UMSARS-II scores and their motor impairment. Comparing the fMRI data between the healthy control group and the MSA group, a bilateral increase in cerebellar cortex activation was detected in the MSA patients. Comparison between the active and sham rTMS groups showed that the cerebellar activation was significantly higher after the real stimulation. This study indicates that HF-rTMS may improve the motor deficits, accompanied by an increased activation of the cerebellum after motor cortex stimulation.

Taken together, these results suggest that HF-rTMS targeting the left M1 probably leads to a significant improvement on motor dysfunction in MSA. Increased activation of the cerebellar cortex as shown with fMRI could correlate with the clinical improvement. However, double-blind studies with larger cohorts are needed, in order for these results to be confirmed and replicated.

## 4. Corticobasal Degeneration (CBD)

The only study investigating the therapeutic role of rTMS in CBS was conducted by Shehata et al. [56]. Twenty-six CBS patients were enrolled in the study and were followed for 12-18 months. A combination of rTMS, pharmacotherapy, rehabilitation therapy, and injection of botulinum toxin was applied. The akinetic-rigid syndrome and cognitive dysfunction were the predominant symptoms for the majority of the subjects. LF-rTMS (1 Hz, 90% of MT intensity) was applied to all patients targeting the contralateral motor cortex of the more damaged side, with one session, 3 times a week for 1 month, every 3 months. The subjects were assessed using a variety of clinical scales and were evaluated every 3 months. In short, after 3 months, the UPDRS, caregiver burden, and quality of life were improved, while cognitive functions remained stable, and this improvement was detected up to 18 months later. *Τ*he lack of control subjects and a possible placebo effect are the main limitations of the study, implying that more clinical trials, sham controlled, randomized, and double-blinded are necessary to elucidate the results of LF-rTMS or other forms of rTMS in CBS therapy.

## 5. Lewy Body Dementia (LBD)

Due to the similarities between LBD and PD and other dementias where rTMS has shown its potential, rTMS has long been insinuated as a possible therapeutic option for LBD [57]. However, there is only one trial assessing rTMS in LBD therapy, focusing on depressive symptoms. In this study, 6 LBD patients with drug-resistant depression were assessed after rTMS intervention. Daily sessions of LF-rTMS (1 Hz, 110% of MT intensity) targeting the right DLPFC and HF-rTMS (10 Hz, 100% of MT intensity) targeting the left DLPFC were applied for ten days. Hamilton Depression Scale (HAL-D) was used for evaluation at baseline and after the intervention showing a significant attenuation of depressive symptoms [58].

## 6. Ongoing Trials

Searching the clinicaltrials.gov website (last accessed on the 24th of November 2021) with the keywords “PSP” and “rTMS”, we came up with 4 studies. Of these, the NCT02236832 study applies rTMS only on healthy participants as a control group and was thus not further read. A cross-over sham-controlled study (NCT04222218), lastly updated in January 2020,will apply cerebellar rTMS-theta burst to PSP patients, assessing its efficacy in postural instability using wearing sensor technology, and has been listed as completed since November 2019, though no results have been made available. Similarly, the NCT01174771 is also listed as complete since February 2012 and was lastly updated in May 2014. This pilot study investigates the potential benefits of the application of rTMS in PSP and CBD patients. This trial proposes that HF- and LF-rTMS targeting motor and prefrontal cortical regions in PSP and CBD patients respectively, may ameliorate motor and cognitive dysfunction; however, no results have been published yet. The NCT04468932, lastly updated in July 2020, investigates the effects of rTMS on motor control in PSP. This study is aimed at proving that cerebellar inhibition via cerebellar LF-rTMS will decrease postural instability in patients with PSP by increasing functional connectivity between the cerebellum, thalamus, and primary motor cortex.

Regarding the research for the studies using rTMS in MSA patients, 2 ongoing trials were found via our search. The NCT04595578, lastly updated in October 2020, applies a combination treatment with cerebellar rTMS and physical therapy (PT) in patients with MSA-C and spinocerebellar ataxia. This pilot study investigates the efficacy and the safety of the combined application of cerebellar rTMS and PT, contrary to the single PT therapy (sham rTMS intervention) in MSA-C patients. However, no results have been published yet. A randomized trial NCT04313530, lastly updated in March 2020, investigates the mechanism and effect of rTMS intervention in MSA patients with fatigue. The researchers' anticipation is that after rTMS there will be a decrease of fatigue in MSA patients, based on the hypothesis that fatigue in MSA may be associated with an altered default mode network and sensorimotor network connectivity.

## 7. Discussion and Conclusions

The majority of rTMS studies on parkinsonism focus on PD. This is reasonable considering the high frequency of this degenerative disease, but the small number of studies on atypical parkinsonian disorders (APDs) highlights the need for additional research regarding these diseases, as they also affect numerous individuals and may ultimately be more debilitating than PD, given the lack of effective treatments.

Regarding PSP, most studies indicated that cerebellar rTMS exerted positive effects, improving postural instability and speech impairment [34–36]. This could be the reflection of improvement in cerebellar-brain inhibition, as Dale et al. [35] and Brusa et al. [34] even quantified and used as an outcome measure for their study, based on studies revealing its diminishing in the setting of PD and PSP [34, 35]. CBI is a physiological cortical inhibition by cerebellar Purkinje cells, crucial for proper motor control. TMS studies revealed that stimulation over the cerebellum recruits the cerebello-thalamo-cortical pathway and restores CBI [59], possibly explaining the amelioration of kinetic parameters shown in cerebellar rTMS studies in PSP. In fact, the study by Brusa et al. [34] showed that CBI was the only cerebellocortical functional connectivity index improved upon cerebellar rTMS. However, only one double-blinded study was available in this domain, so evidence is preliminary at best.

Furthermore, rTMS application over the motor area and the DLPFC showed beneficial effects in motor and depressive symptoms, respectively [38–40]. Nevertheless, several questions arise, which still remain unanswered. In almost all of the aforementioned studies, the PSP patient groups almost exclusively included the Richardson's syndrome subtype of PSP. The update of the clinical diagnostic criteria for PSP in 2017 emphasized the large phenotypical heterogeneity of PSP. Richardson's syndrome appears as only one type of the ten possible PSP phenotypes. There are no clinical trials examining the effect of rTMS on the rarer SP phenotypes. Only Nishida et al. included six patients with a different variant, the PSP-pure akinesia with gait freezing (PSP-PAGF). As such, more studies are needed, to evaluate the efficacy of rTMS to the whole phenotypical spectrum of PSP. Additionally, conflicting results have arisen due to both LF and HF protocols giving positive results. Regarding the motor symptoms, reduced intracortical inhibition has been highlighted as a feature of PSP [44], so LF protocols, which induce inhibitory changes, may hold more meaning to be explored in the future. Besides, the two studies that applied HF-rTMS and reported positive results [34, 35] showed that these were either short-lasting or insignificant in the various subitems. Finally, Madden et al. [41] reported a case of tDCS improving language deficits in a PSP patient. Albeit not rTMS, this study is important in bringing forward the potential of noninvasive brain stimulation as an effective modality in neurodegenerative diseases and PSP in particular.

The rTMS studies regarding the cerebellum in MSA have not aided in pinpointing a certain direction this far. The few available studies have been vastly heterogeneous, and regarding the cerebellum, both LF and HF protocols over the same area seem to be beneficial, one regarding motor and the other cognitive performance [47, 60]. This seems heavily counterintuitive and further raises questions of erroneous methods in the studies. In MSA, the cerebellum seems to be affected in a way that is similar to PSP; reduced physiological cerebellar inhibitory inputs give way to motor disorders. In this sense, HF protocols, increasing this input, should be able to present better results, as shown in the study addressing motor deficits. The reasons behind LF-protocol seemingly producing cognitive benefits remain unclear; it could be the case that different circuits are involved in each pathology but without further studies to counter or corroborate the aforementioned results; one cannot reach any conclusions. On the contrary, the results of two sham-controlled studies involving the left motor cortex have provided consistent positive results, with implication of the cerebellum as well [48, 61]. However, both of them were conducted by the same group and were not double-blinded.

The search for studies on rTMS and CBD or LBD yielded only two trials involving patients of these degenerative disorders. First, Shehata et al. [56] studied the efficacy of LF-rTMS to twenty-six CBS patients. According to their results, many disease parameters were improved after three months, and the improvement was maintained for more than a year postintervention. The rationale of this study in applying LF-rTMS lay in studies showing reduced cortical inhibition in LBD and in previous studies of rTMS over the motor cortex of PD patients yielding positive results. This train of thought is useful, in drawing inspiration for the already lain road of PD, and more studies in this direction are more than encouraged.

Of note, this is the only study in the mentioned literature that followed the patients for 18 months and could draw conclusions on the long-term results of the intervention. The duration and the persistence of the beneficial effects of a therapeutic intervention are of major importance when assessing a therapeutic option, and more research is needed regarding near-transfer effects of rTMS in APDs and the longitudinal observation of possible rTMS benefits. It will be even more interesting to see whether rTMS is even capable of slowing the progression of some of these diseases and gain a preventative, rather than a solely therapeutic role.

Future studies should address some issues mostly concerning the study design. Large studies with big cohorts are not easy to be organized, as a lack of equipment and qualified research staff is often encountered. A multicenter study design could gather larger samples of patients, and consequently, more accurate results could be obtained. Sham-controlled studies must be preferred, so that the placebo effect may be controlled for. Some of the mentioned trials did not apply sham stimulation, driving to a lower quality of their study. Dale et al. [35], who investigated cerebellar rTMS effects in two PSP patients, used sham stimulation, though a different sham stimulation was applied on each of the patients. One of them could understand the sham intervention since this superficial stimulation could not produce the same sensation over the head and neck area as the real one. Naturally, this placebo effect raises doubts on the trial findings and highlights the need for proper methodology.

The application of rTMS in earlier disease stages is another issue that needs to be discussed. For instance, regarding AD, a common degenerative disorder combined with dementia, the excitant literature shows that patients in earlier stages had responded better after treatment with rTMS [32, 62, 63]. This phenomenon could be explained from the smaller degree of brain atrophy, contributing to better responsiveness to rTMS [64]. An early diagnosis of APDs would enable the earlier application of rTMS with probably better modulation effects, but the great variety of phenotypical expression of these disorders and their lower prevalence contribute to a difficult early differential diagnosis. Nowadays, there are important scientific attempts towards reaching an accurate and early diagnosis of APDs, using updated clinical criteria, functional imaging, and nuclear medicine. As such, future trials could attempt to assess the effect of rTMS therapy on early stages of these disorders or compare its efficacy between earlier and more advanced stages.

In conclusion, particularly because of the limited pharmacologic and nonpharmacologic treatment options for patients with APDs, rTMS is a promising tool for therapy. However, the determination of the exact therapeutic protocols still has a long way to go due to the lack of large-scale trials, driving to the urgent need of high quality clinical studies, providing strong evidence on the persistence and reproducibility of the observed beneficial effects.

## Figures and Tables

**Table 1:** Studies assessing the effects of rTMS in PSP.

Author, year	Type of study	Study design	Results
Brusa et al. [34]	Prospective cohort study/open label	(i) 10 PSP patients, 10 PD patients, 10 HC (ii) Lateral cerebellum bilaterally (iii) ITBS protocol (3 50 Hz pulses, repeated at a rate of 5 Hz, 20 trains of 10 bursts in 8 s intervals, 600 pulses, 80% of AMT intensity) for two weeks (iv) Assessment at baseline and after 2 weeks via rs-fMRI and PSP-RSc	(i) Clinical improvement (dysarthria, gait) and a parallel enhancement in functional connectivity between the cerebellar hemisphere and motor cortex (ii) No adverse events
Dale et al. [35]	2 PSP study cases/sham controlled	(i) 2 PSP patients (ii) Cerebellum (iii) RTMS (10 Hz, 4.000 pulses, 4 seconds on, 8 seconds off, 100 trains, 90-110% of RMT intensity) 10 days active 10 days sham stimulation, separated by a month (iv) Assessment at baseline and immediately after treatment	(i) CBI increased/improvement in stability and speech (ii) Pending tolerability
Pilotto et al. [36]	Double blind/sham controlled	(i) 20 PSP patients (ii) Cerebellum (iii) TBS (3 50 Hz pulses repeated at a rate of 5 Hz, 20 trains of 10 bursts in 8 s intervals, 600 pulses, 80% RMT intensity) (iv) Clinical evaluation (Tinetti test, the Short Physical Performance Battery (SPPB), the Timed Up and Go test, and the Functional Reach test (FR)) and static balance assessed before and after active and sham stimulation, inertial sensor unit (IMU) processing accelerator signals	(i) Beneficial effect on postural instability and improvement in area, velocity, acceleration, and jerkiness of sway (ii) No adverse events
Santens et al. [37]	Prospective cohort study/open label	(i) 6 PSP patients (ii) Lower limb motor area (iii) RTMS (10 Hz, 1.000 pulses, 5 seconds on, 55 seconds off, 20 trains, 80% of MT intensity) for 5 consecutive days (iv) Assessment with PSP-RSc at baseline and after 5 days	(i) Improvement on the gait and midline symptoms (ii) No adverse events/discomfort during the stimulation
Nishida et al. [38]	Prospective cohort study/open label	(i) 7 PSP patients (ii) Supplementary motor area (SMA) (iii) RTMS (5 Hz, 500 pulses,10 trains, 10 seconds on, 110% of RMT intensity) for 10 days (iv) Assessment using PSP-RSc at baseline and immediately after treatment	(i) Improvement of the PSP-RS by 7 points (ii) No adverse events
Major et al. [39]	1 PSP case study/open label	(i) 1 PSP patient (ii) Bilateral motor cortex area (iii) LF-rTMS (1 Hz, 80% of RMT intensity) 20 min per day, for five consecutive days (iv) Assessment using mechanometry and goniometry at baseline and after 5 days	(i) Increase in the range of motions and in the muscle forces (ii) No adverse events
Boulogne et al. [40]	1 PSP case study/open label	(i) 1 PSP patient (ii) Right dorsolateral prefrontal cortex (DLPFC) (iii) LF-rTMS (1 Hz, 6 trains, 1 min on–30 sec off, 120% of RMT intensity) (iv) Assessment at baseline and immediately after treatment via the Montgomery Asberg Depression Rating scale (MADRS), the State-Trait Anxiety Inventory (STAI), the Lille Apathy Rating Scale (LARS), and the Global Assessment of Functioning (GAF) Scale. The PSP-RSc and the MoCA were assessed before and after the rTMS treatment	(i) Relieve depression/MADS and STAI scores decreased; the LARS and GAF scale scores increased after rTMS (ii) No adverse events
Madden et al. [41]	1 PSP study cases/sham controlled	(i) 1 PSP patient (ii) Left dorsolateral prefrontal cortex (DLPFC) (iii) TDCS (iv) Assessment at baseline and immediately after treatment via language tasks	(i) Improve phonemic fluency and action naming (ii) No adverse events

**Table 2:** Studies assessing rTMS in MSA.

Author, year	Type of study	Study design	Results
Liu et al. [46]	Prospective cohort study/open label	(i) 9 MSA patients (ii) M1 bilaterally lateral cerebellum bilaterally (iii) HF-rTMS (5 Hz, 2000 pulses, 500 for each target, 50 trains, 100% of RMT intensity) for 5 days (iv) Assessment at baseline and after 5 days via fMRI and UMSARS	(i) Improved motor control and increased resting-state complexity within the motor cortex (ii) No adverse events
Yildiz et al. [47]	Prospective cohort study/open label	(i) 12 MSA-C patients, 5 AD patients, and 9 healthy controls (ii) Lateral cerebellum (iii) LF-rTMS (1 Hz, 600 pulses, 90% of RMT intensity) (iv) Evaluation in 2 different sessions in the same day using a computerized reaction time (RT) task and SAI responses	(i) Improvement in SAI deficits, improvement in post-rTMS RT values in the MSA-C group in contrast with the pre-rTMS RT (ii) No adverse events
Chou et al. [48]	Randomized/double-blind/sham controlled study	(i) 21 MSA patients (ii) Left M1 (iii) HF-rTMS (5 Hz, 1000 pulses, 10 trains, 110% RTM intensity) for 10 days, one session per day (iv) Assessment at baseline and after 5 and 10 days via fMRI and UMSARS-II	(i) Improvement of motor symptoms, increased brain functional connections in the active rTMS group (ii) No adverse events
Wang et al. [49]	Prospective cohort/sham controlled study	(i) 15 MSA patients and 18 healthy controls (ii) Left M1 (iii) HF-rTMS (5 Hz, 1000 pulses, 10 trains, 110% RTM intensity) over the left M1 over 2 weeks (iv) Assessment at baseline and after 5 and 10 days via fMRI and UMSARS-II	(i) Improvement of motor symptoms, increased activation in the bilateral cerebellum in the active rTMS MSA group (ii) No adverse events

**Table 3:** Studies assessing rTMS in CBD and LBD.

Author, year	Type of study	Study design	Results
Shehata et al. [56]	Prospective cohort study/open label	(i) 26 CBS patients (ii) Motor cortex contralateral to the more affected side (iii) LF-rTMS (1 Hz, 90% of MT intensity), a session 3 times a week for 1 month, every 3 months (iv) Assessment at baseline and every 3 months over 18 months via UPDRS, Addenbrooke's cognitive examination (ACE-R), Unified Dystonia rating scale (UDRS), HRQoL, caregiver burden questionnaire and videotaping	(i) The UPDRS, caregiver burden, and quality of life were improved after 3 months (ii) No adverse events
Takahashi et al. [58]	Prospective cohort study/open label	(i) 167 patients with mood disorder, 6 DLB patients received rTMS (ii) DLPFC bilaterally (iii) LF-rTMS (1 Hz, 110% of MT intensity) over the right DLPFC and HF-rTMS (10 Hz, 100% MT intensity) for the left DLPFC daily for ten days (i) Assessment at baseline and after 10 days via the HAM-D	(i) Improvement of depressive symptoms (ii) No adverse events
